# Primary Care Practice Transformation and the Rise of Consumerism

**DOI:** 10.1007/s11606-016-3946-1

**Published:** 2017-02-27

**Authors:** William H. Shrank

**Affiliations:** UPMC Health Plan, U.S. Steel Tower, 55th Floor, 600 Grant Street, Pittsburgh, PA 15219 USA

## Abstract

Americans are increasingly demanding the same level of service in healthcare that they receive in other services and products that they buy. This rise in consumerism poses challenges for primary care physicians as they attempt to transform their practices to succeed in a value-based reimbursement landscape, where they are rewarded for managing costs and improving the health of populations. In this paper, three examples of consumer-riven trends are described: retail healthcare, direct and concierge care, and home-based diagnostics and care. For each, the intersection of consumer-driven care and the goals of value-based primary care are explored. If the correct payment and connectivity enablers are in place, some examples of consumer-driven care are well-positioned to support primary care physicians in their mission to deliver high-quality, efficient care for the populations they serve. However, concerns about access and equity make other trends less consistent with that mission.

## THE PRIMARY CARE LANDSCAPE

The practice of primary care is undergoing a rapid transformation. The movement towards value-based purchasing and the broad adoption of alternative payment models is driving an evolution in the role of the primary care physician. Primary care practices are now expected to focus on the management of the health of populations, higher-quality care, improved prevention, and a more careful consideration of resource utilization and expenditures. Whereas physicians were previously reimbursed solely for the services they delivered to patients at the time they visited the doctor’s office, physicians are increasingly rewarded for the preventive services delivered to patients between office visits and for the elective or unplanned services that are not delivered to patients. As described by Ellner and Phillips in this JGIM symposium, these trends are likely to accelerate.^1^ In this new environment, primary care physicians in some settings are receiving capital from payers to support ancillary staff and infrastructure to better care for patients with chronic conditions, assess patient risk, and focus resources on the patients who need them most.

In this context, attracting patient panels that are loyal, adherent, and engaged is a central theme for primary care physician success. Physicians will have a greater opportunity to deliver high-quality care and promote value when their patients show up for their appointments and preventive tests, adhere to their medications, and endorse healthy lifestyles. This creates a tension for physicians. Physicians need to promote deep, trusting, personal relationships with patients. At the same time, as discussed elsewhere in this JGIM symposium, primary care physicians must leverage ancillary staff to operate at the top of their licenses,^1^ apply systematic screening, analytics, and technical tools^2^ to guide resource use, and promote evidence-based utilization of health services,^3^^,^ 4 even when patients request otherwise.

## THE RISE OF CONSUMERISM

This tension for primary care physicians is exacerbated by the rise of consumerism in healthcare. Patients are demanding to be much more active participants in their care decisions. With the emergence of insurance exchanges introduced under the Affordable Care Act, Americans are playing a greater role in selecting their own insurance products, and are more informed about the coverage process. The information superhighway offers patients much greater opportunity to search for etiologies of symptoms and potential therapies.^5^^,^ 6 Patients experiencing symptoms or diagnosed with a health condition are increasingly seeking information and emotional support from peers on social media sites.^7^^–^ 9 Patients frequently request specific pharmaceutical treatments they see advertised in the lay media.^10^ More informed consumers may challenge primary care physicians in their efforts to guide patients through evidence-based algorithms for care.

More importantly, patients are expecting more convenient care and more responsive physicians. Patient ratings of quality care, which often emphasize trust in physicians, health-related communication, and rapport building, sometimes fail to correlate with clinical indicators of quality, suggesting an opportunity to improve the patient experience and perceptions of care.^11^^,^ 12 One study found that 40% of the variation in patients’ overall rating of care was related to the time they spent waiting in the waiting room.^13^

For primary care physicians, delivering service that meets the demands of the healthcare consumer might be expected to drive up the cost of healthcare. Yet, by failing to offer consumer-centric service, physicians may alienate the most “engage-able” patient populations that must be the focus of accountable primary care and the basis of efforts to manage populations. Through this lens, several recent consumer-driven trends in care delivery are considered below.

## THE EXPANDING ROLE OF RETAIL HEALTHCARE

In efforts to address the demand for convenient care, there has been a dramatic expansion in the delivery of primary care services in retail settings. As of 2015, there were over 2000 retail clinic sites in the United States.^14^ These clinics generally offer acute services for a limited number of diagnoses, and follow strict evidence-based management guidelines. The evidence describing the quality of care delivered at retail clinics to date has been reassuring. Multiple studies have shown that for common acute illnesses such as upper respiratory tract infection, urinary tract infection, or pharyngitis, the care provided at retail clinics is as good as or better than care delivered in ambulatory or emergency room settings.^15^^,^ 16 Moreover, these studies have shown that strict guideline adherence is associated with a reduction in the inappropriate use of antibiotics, dispelling a common concern about conflicts of interest arising from care delivered in a retail pharmacy setting.^17^

The evidence regarding the relationship between retail clinics and total healthcare costs is less clear. There is no question that care delivered at retail clinics is less costly than that in other settings.^18^ However, there have been conflicting results in studies evaluating whether retail clinics serve as a substitute for more costly sites of care, or as a way to provide additional supply for perceived unmet demand, increasing overall utilization of healthcare services.^19^^,^ 20 Nonetheless, many primary care physicians appreciate the opportunity to refer their patients to retail clinics for simple acute problems on weekends and evenings as an alternative to expensive emergency room care. Primary care physicians who are taking on financial risk for the management of their populations have been far more likely to partner with retail clinics. Alternatively, other physicians, more commonly in the fee-for-service environment, consider retail clinics a source of competition. Retail clinics are seen as “skimming” the easier cases and leaving the more time-consuming patients for the primary care physician, adding new challenges to their workflow as they coordinate care from another site without reimbursement for those efforts.

Several competing models of retail care have emerged that highlight different relationships and value propositions for primary care practitioners. MinuteClinics, retail clinics at CVS/pharmacy locations, propose to serve as extenders of primary care rather than substitutes. Staffed by nurse practitioners, they do not care for patient panels.; rather, they manage acute conditions and return patients to their primary care physicians or help patients without primary care physicians find a medical home. MinuteClinics commit to sharing data about encounters with a patient’s primary care physician in order to maintain continuity of care. Walgreens operates some clinics that employ a model very similar to that seen at CVS, and is also experimenting with a competing model in which physicians from health systems staff the clinic and refer patients without primary care physicians into their health systems. Several partnerships with large health systems, such as Advocate in Chicago, have been announced.^21^

Both of these models leverage convenient sites of care as low-cost extensions of the primary care physician. Neither aspire to be substitutes for the primary care physician or competitors to the typical primary care model. As such, the existing movement towards local, convenient care in retail settings does not seem to be a major disruption in the primary care model. CVS and Walgreens are working to improve electronic connectivity with primary care physicians; these trends should help retail clinics serve as a mechanism for supporting consumer choice. As primary care physicians take more financial risk for the health and costs of the patients they serve, and yesterday’s profit centers become today’s cost centers, these types of partnerships with retail care should help to deliver patient-centered care without driving increased costs.

However, electronic connectivity is far from complete, and primary care physicians continue to be responsible (and are not compensated) for the interpretation and coordination of care across settings. More importantly, if retail clinics were to challenge the existing model, and did aspire to take over the role of the primary care physician (e.g. provide continuous, comprehensive, coordinated, 24-hour access to primary care),^1^ this could represent a major disruption in the existing primary care model. Retail primary care could address a need resulting from primary care physician shortages and enhance access to essential care. At the same time, the emergence of retail primary care would create competition with existing primary care physicians, and would raise important new questions about quality and care coordination. In this rapidly evolving marketplace, continued attention to these emerging models will be essential.

## CONCIERGE AND DIRECT CARE

Some primary care physicians are meeting patient demand for convenience and greater attention by contracting directly with patients, either via “concierge care,” where the patient pays a retainer fee that provides for highly personalized, round-the-clock access but where the physician still bills the patient’s insurance for services, or “direct care,” where the patient pays out-of-pocket for all services, bypassing insurance altogether.^22^^,^ 23 Concierge practices typically charge $1500 or more a year, with elite practices charging as much as $25,000 annually.^24^ A 2013 survey found that approximately 6% of physicians were in concierge or cash-only practices,^25^ with surprisingly low levels of attrition during the economic recession.^24^ Advocates of direct care believe that such arrangements improve quality, as they remove insurers from the authorization process and offer physicians ample time to meet the needs of willing patients.

The appeal of these models for primary care physicians is clear. The models tend to be highly lucrative, with a more manageable work schedule, less paperwork, and greater flexibility and time to care for their patient panels.^22^^,^ 26 Panels tend to be much smaller than those of typical primary care physicians (400–600 patients as compared to a typical 2500-patient primary care panel).^26^ In some ways, this model affords physicians the time and resources to concentrate on health promotion in their panels, often engaging health coaches and enforcing preventive screening and high-touch interactivity with the panel of patients served. That additional time can permit careful diagnostic scrutiny including careful history taking and physical examination—applied both to the ill patient with undifferentiated illness and to the worried well.

However, this model seems to run counter to the overall mission of payment and delivery reform. Such exclusive practices would be expected to further reduce the already limited supply of primary care physicians that do take health insurance, potentially limiting access to primary care for those without the means to contract directly. Moreover, this model may be expected to exacerbate disparities in care, as the most vulnerable will be most likely to face access issues. The result could be a tiered system of primary care, where those physicians who do not contract directly with patients would care for a sicker and more vulnerable population, further challenging their mission to manage the health of the populations they serve. Nevertheless, the popularity of concierge care and direct primary care suggests that patients with the financial wherewithal are willing to pay for access to high-quality primary care; these trends underscore patients’ interest in a more meaningful relationship with their primary care physician. And from the perspective of the primary care physician, there is a clear desire to get out from under the yoke of insurance paperwork, documentation requirements, and time pressure. Absent issues of physician supply and equity, these models are attractive to many patients and physicians alike, and have implications for the future design (“reinvention”) of primary care.

To date, little evidence is available to measure the effect of these models on quality or costs of care among patients who participate, or quality and access for those who do not.^22^ However, the relatively modest uptake of these models suggests that the level of disruption thus far is low. These practices tend to be offered by individual practitioners or small groups, and not by integrated health systems. As physicians increasingly join larger, more integrated practices, expansion of this model would be expected to decelerate. Careful attention to the rates of uptake of direct contracting and concierge care is essential in assessing whether any markets reach a “tipping point,” where these models have sufficient presence to warrant studies within geographic regions to evaluate the impact on participating panels of patients as well as the populations that are not served by these models.

## HOME-BASED DIAGNOSTICS AND CARE

Home-based diagnostics, designed to meet consumer demand for the convenience of self-testing and immediate access to test results, are becoming increasingly popular, supported by rapid innovation in technology.^27^^,^ 28 The global market for home-based diagnostic testing is projected to reach nearly $37 billion by 2021.^29^ The long-standing availability of home testing for pregnancy and blood glucose levels has given rise to point-of-care lab testing for infectious diseases including hepatitis C and HIV and chronic conditions such as hypercholesterolemia or anemia, and even symptomatic problems such as allergy.^28^ Of course, the tests must be dependable, and the recent devaluation of Theranos highlights the important role played by the FDA in ensuring that the tests are accurate and can be interpreted by consumers.^30^ Nonetheless, home-based testing for conditions like high blood pressure and blood glucose are now mainstays of chronic disease management and provide essential data to physicians about a patient’s condition between office visits.

Home-based diagnostics can also serve as a meaningful set of tools to promote timely and cost-effective diagnosis. Tests should be far less expensive when administered at home, eliminating the overhead and assorted marginal costs of an office visit. Evidence characterizing the potential savings in total healthcare costs that these diagnostics represent is currently limited. However, the value proposition is intuitive, and if evidence is developed, payers will likely provide greater coverage for the use of home-based diagnostics. And as the proliferation of wireless technology continues, the opportunity for home-based diagnostic results to be electronically connected with physicians also comes into focus. New technology that allows a consumer to perform an ECG at home with an iPhone underscores the potential for connected physicians to diagnose and manage costly and increasingly complex conditions in lower-cost and more convenient settings.^6^

Considering the mounting business case for device manufacturers, insurers, and risk-bearing health systems to expand the use of home-based diagnostics, and the demand from informed patients for rapid and convenient answers to clinical questions, there seems to be a straightforward pathway for these tests to play a disruptive role in the delivery of primary care. Thus primary care physicians may consider the patient’s home as an extension of the examination room—a place where a history may be taken and a definitive diagnostic test performed, and where convenient and comprehensive primary care can be delivered without sacrificing quality. As physicians take on greater financial risk for total healthcare costs and increasing responsibility for the health of populations they serve, the thoughtful use of home-based diagnostics should be fully aligned with the goals of payment and delivery reform. Further study clarifying the business case for expanding coverage and investing in connectivity should be performed to allow for rapid scaling of successful models.

Beyond diagnostics, home-based primary care is also gaining popularity. The recent release of results from the Centers for Medicare & Medicaid Services (CMS) Independence at Home Demonstration indicates that for the most vulnerable patients, home-based care can improve outcomes and reduce total healthcare costs while improving the patient experience.^31^ The reach for home-based delivery of primary care will remain limited, however, as only the most vulnerable and sedentary patients are likely to be eligible candidates, due to the cost of care. Moreover, transportation can be a central challenge for patients, and is a leading reason that patients do not show for primary care visits.^32^^,^ 33 Partnerships with Uber and Lyft have been announced by multiple physicians across the U.S. to provide transportation and enhance access to care.^34^^,^ 35 As physicians assume greater responsibility for their patients’ health, addressing the key barriers to care and improved health, such as transportation, may be critical. The movement towards a greater amount of connected care in the home seems inevitable, and likely will include a blend of telehealth, home-based delivery, and greater connectivity.^2^ A rich discussion of telehealth in primary care is presented by Young and Nesbitt elsewhere in this issue.^2^

## ENABLERS OF THE RISE OF CONSUMERISM IN PRIMARY CARE

Primary care physicians seeking to transform their business model to one of value-based purchasing face a seeming challenge from the new demands of an empowered healthcare consumer. The tension between the need to manage costs and population health and the need to deliver care that is tailored to the convenience of individual patients is evident. The marketplace is adapting to meet the needs of consumers, as demonstrated by the rise of retail care, home-based care, and direct contracting with patients. The extent to which primary care physicians can sort through these trends and identify opportunities to deliver more patient-centered, convenient care, while also achieving the ultimate goal of providing better care and at a lower cost, will be critical.

While retail care and home-based diagnostics and care may appear to challenge the existing primary care model, if employed correctly, they should be able to promote more rapid practice transformation. Retail and home-based care, when delivered in coordination with an engaged primary care practice, should offer more convenient and lower-cost options for physicians and patients, promoting better care at lower cost. However, the movement towards direct contracting for primary care runs counter to the premise that the healthcare system has a broader responsibility to consider resource allocation and the health of all patients in the care models that are adopted. The extent to which primary care physicians embrace their role in managing the health of the population and delivering equitable care remains to be seen. The conflict that arises between the direct care model and the goals of payment and delivery reform are likely irreconcilable.

Two essential enablers for consumer-centric primary care are connectivity infrastructure and payment. Retail care and home-based diagnostics and care will succeed only if patients, ancillary physicians, devices, and primary care practices can communicate when patients are not in the office. This level of connectivity will allow consumer-based care to be coordinated, and not a source of fragmentation and inefficiency. At the same time, payers must be willing to entertain new evidence about consumer-centric models of care and to provide coverage for services that promote convenient, lower-cost care in non-traditional settings. Failing to do so may, paradoxically, slow primary care transformation and lead to greater fragmentation and higher costs. In the presence of connectivity and supportive payment mechanisms, the rise of consumerism and the rapidly innovating marketplace should drive more personalized provider–patient relationships and deeper engagement in care. These will be a key focus as physicians transform their practices to deliver better and more equitable care for populations and to reduce unnecessary healthcare costs.
